# STITCH 5: augmenting protein–chemical interaction networks with tissue and affinity data

**DOI:** 10.1093/nar/gkv1277

**Published:** 2015-11-20

**Authors:** Damian Szklarczyk, Alberto Santos, Christian von Mering, Lars Juhl Jensen, Peer Bork, Michael Kuhn

**Affiliations:** 1 Institute of Molecular Life Sciences, University of Zurich and Swiss Institute of Bioinformatics, Winterthurerstrasse 190, 8057 Zurich, Switzerland; 2 Novo Nordisk Foundation Center for Protein Research, Faculty of Health and Medical Sciences, University of Copenhagen, 2200 Copenhagen N, Denmark; 3 European Molecular Biology Laboratory, Structural and Computational Biology Unit, Molecular Medicine Partnership Unit, Meyerhofstrasse 1, 69117 Heidelberg, Germany; 4 Max-Delbrück-Centre for Molecular Medicine, Robert-Rössle-Strasse 10, 13092 Berlin, Germany; 5 Max Planck Institute of Molecular Cell Biology and Genetics, Pfotenhauerstrasse 108, 01307 Dresden

## Abstract

Interactions between proteins and small molecules are an integral part of biological processes in living organisms. Information on these interactions is dispersed over many databases, texts and prediction methods, which makes it difficult to get a comprehensive overview of the available evidence. To address this, we have developed STITCH (‘Search Tool for Interacting Chemicals’) that integrates these disparate data sources for 430 000 chemicals into a single, easy-to-use resource. In addition to the increased scope of the database, we have implemented a new network view that gives the user the ability to view binding affinities of chemicals in the interaction network. This enables the user to get a quick overview of the potential effects of the chemical on its interaction partners. For each organism, STITCH provides a global network; however, not all proteins have the same pattern of spatial expression. Therefore, only a certain subset of interactions can occur simultaneously. In the new, fifth release of STITCH, we have implemented functionality to filter out the proteins and chemicals not associated with a given tissue. The STITCH database can be downloaded in full, accessed programmatically via an extensive API, or searched via a redesigned web interface at http://stitch.embl.de.

## INTRODUCTION

The role of small molecules in biological systems can be understood only in the relation to the function of the targeted biomolecules, which, in turn, is largely defined by their interaction partners (1–3). The role of the interaction network is even more prominent in the area of the drug development, since diseases are often a consequence of multiple changes in the same pathway or protein complex (4,5). Taking into account the neighborhood of the targeted proteins and the topology of the network itself can lead to a better understanding of a drug's cellular impact (6,7). Furthermore, as only a subset of all proteins are viable drug targets (8), most therapeutics target proteins in the network vicinity from more prospective, but undruggable, proteins (7). Several databases provide proteome-wide protein–chemical interactions (9–11) and several other (12–14) put protein–chemical interactions in the context of protein–protein interaction networks, which is essential for effective *in silico* drug discovery.

A drug's impact on the organism and its efficacy depend on its engagement with the targeted proteins and the extent to which it disrupts the protein–protein and protein–chemical interaction network (7,15). This is related to the concentration of the drug, the strength with which it modulates the activity of the target, and the distribution of target proteins among different tissues (16). To enable the users to rationally select possible drug targets, we have added two new features to STITCH: a new mode that allows users to show known binding affinities between proteins and chemicals, and the ability to filter the network to show only proteins related to a selected tissue.

STITCH, in its fifth release, shares protein space with STRING v10 (17) and now encompasses more than 9 600 000 proteins from 2031 eukaryotic and prokaryotic genomes. Also, its chemical space grew by a quarter compared to the previous version (18), from 340 000 to 430 000 compounds (not including different stereoisomers). STITCH is available through new redesigned web interface at http://stitch.embl.de and via an extensive API that allows programmatic access, including the ability to disambiguate queries, modify all network parameters and generate images. In order to enable large-scale analysis, which may not be feasible through web-interface or API, the precomputed network and the supplementary information are freely available for download.

## SOURCES OF INTERACTIONS

Although there is a plethora of data available from which protein–chemical networks could be derived, their dispersed nature, different precision, name-space and focus make it cumbersome to assemble a full picture of all available knowledge. The STITCH pipeline aggregates high-throughput experiments data, manually curated datasets and the results of several prediction methods into a single global network of protein–protein and protein–chemical interactions. This does not expose the user to the heterogeneity of the underlying data, yet, at the same time, keeps all the primary evidence of the interaction readily accessible.

A large part of the known interactions comes from manually curated datasets such as DrugBank (19), GPCR-ligand database (GLIDA) (20), Matador (21), the Therapeutic Targets Database (TTD) (22) and the Comparative Toxicogenomics Database (CTD) (23), and several pathway databases including the Kyoto Encyclopedia of Genes and Genomes (KEGG) (12), NCI/Nature Pathway Interaction Database (24), Reactome (25) and BioCyc (26). As there can be overlap between different manually curated datasets, we do not consider multiple reports of identical interactions as being independent from each other. Instead, we count redundant interactions only once and do not increase the confidence level. Other large sources of protein–chemical links are the datasets of experimentally validated interactions, which include ChEMBL (27), PDSP *K*_i_ Database (28), Protein Data Bank (PDB) (29) and two high-throughput kinase–ligand interactions studies (30,31). Also in this case, interactions may be reported in different databases and with different binding affinities. To compute the final confidence score, we only take the strongest reported affinity into account.

The sources of verified protein–chemical interactions are complemented by automated text mining and a structure-based prediction method (18). The text-mining pipeline include co-occurrence text-mining and natural language processing of all MEDLINE abstracts as well as available PubMed Central open-access full-text articles (32). The newest addition to the text-mining sources are NIH RePORTER grant abstracts (https://projectreporter.nih.gov/). Considering co-occurring terms, adding the RePORTER data increased the number of high-confidence interactions between human proteins and chemicals from 2740 to 4740. Extensive benchmarking of each data source allows us to provide unified confidence score for every interaction while taking into account the sources’ predicted precision.

## DISPLAY OF BINDING AFFINITIES IN THE NETWORK VIEW

Small molecules that activate or inhibit proteins such as enzymes or receptors are among the most studied classes of exogenous small molecules. In order to assess the effect and confidence of protein–ligand binding, as well as variability in the affinity of known ligands, it is essential to know the binding affinity between the compound and its target. Usually, this binding affinity is quantified as the inhibition constant *K_i_*. In some cases, *K*_i_ values are not available, but other values such as the IC_50_ or EC_50_ (half of the maximal inhibitory concentration) can serve as an approximation. *K*_i_ values of drugs vary greatly, from nanomolar inhibition constants to relatively high values, such as 52 μM between aspirin and cyclooxygenase 2 (27). Therefore, for any given drug, it is not so much the absolute value of the *K*_*i*_, but rather the relative binding affinities that determine the impact on the interaction network.

**Figure 1.**
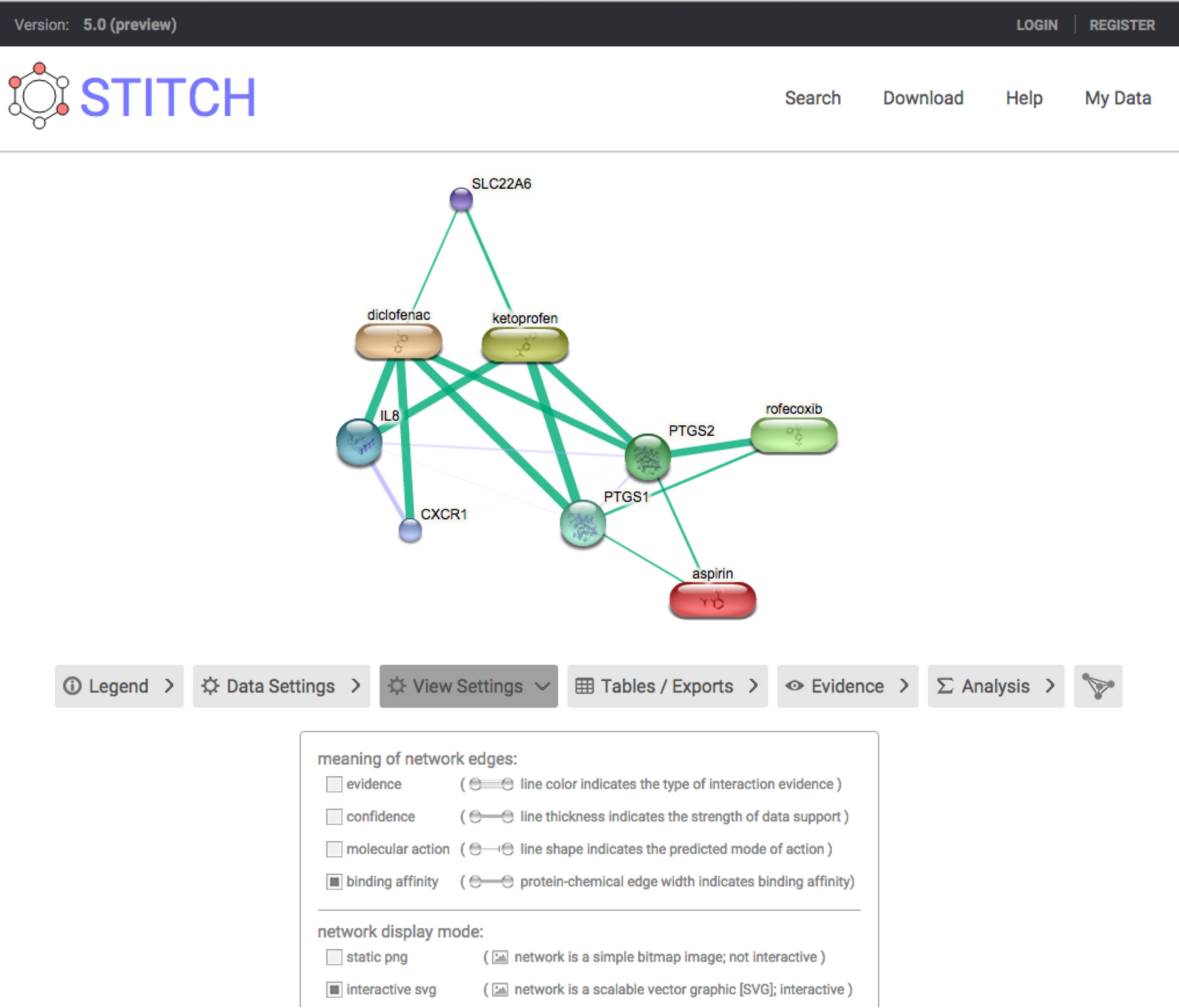
Display of binding affinities. The user interface of STITCH has been updated and the option to scale edge width of protein–chemical interactions according to binding affinity has been added. The shown network of multiple NSAIDs makes their different binding affinities clear: for example, aspirin has relatively low binding affinities, whereas rofecoxib is specifically binding PTGS2.

In previous versions of STITCH, *K*_i_ values from primary sources (27,28) were accessible to the user through the web-interface. In the new release of STITCH, the user can now choose to switch the network view to show the binding affinities of all protein–chemical interactions for which this value is known (Figure 1). This new network view is similar to the STITCH's confidence view: the thickness of the edge between nodes scales with the *K*_i_ value. If a *K*_i_ is not available, EC_50_ or IC_50_ will be used to determine the depicted strength of the interaction. If there are multiple measurements available, the lowest value (i.e. highest reported affinity) will be used to determine the thickness of the edge.

## DATA AND FILTERING FOR TISSUE SPECIFICITY

**Figure 2.**
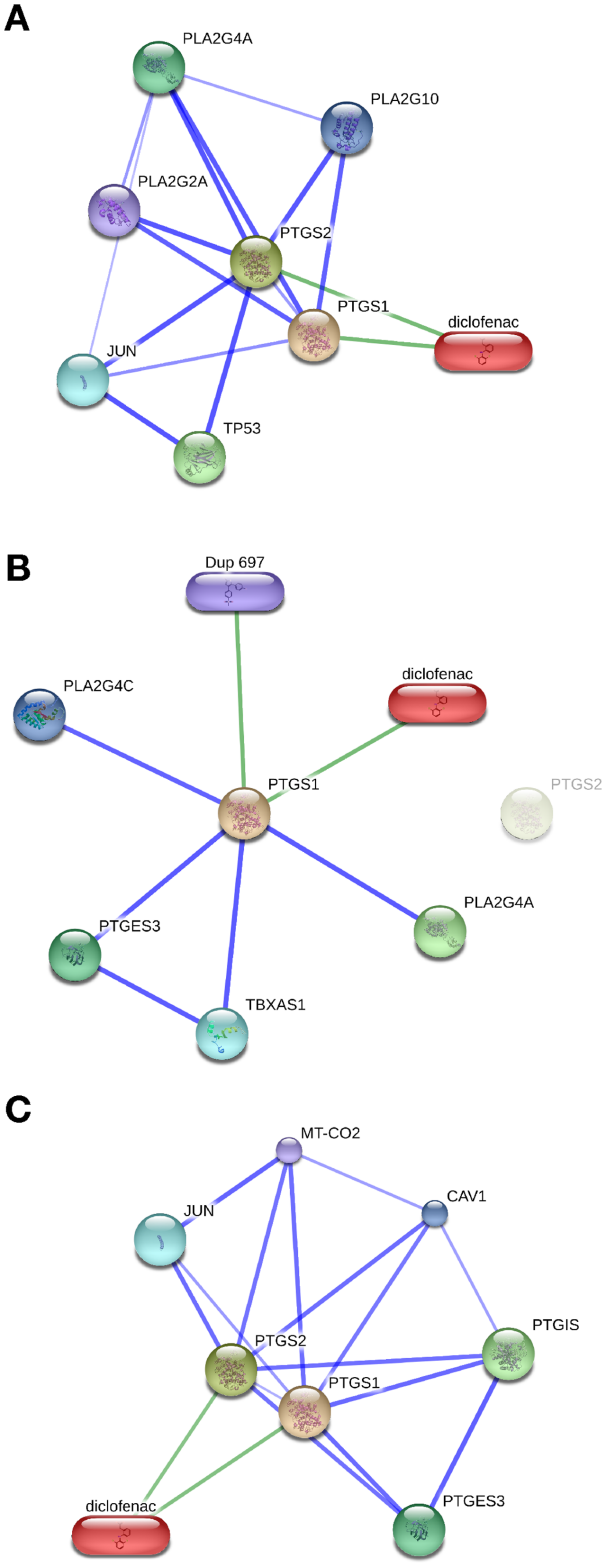
Filtering interaction networks according to tissue expression patterns. (**A**) The interaction network around diclofenac and PTGS1/2 is shown without filtering for tissue expression patterns. In this and the following panels, the top five interaction partners with the highest scores are shown. (**B**) Using the TISSUES resource, only proteins believed to be expressed in blood platelets (with medium confidence, i.e. three stars in TISSUES) become part of the interaction network. For these settings, PTGS2 is not expressed and is therefore shown in a lighter color. (**C**) Expression patterns according to RNA-seq data from the Human Protein Atlas are used to focus on genes expressed in smooth muscle. Confidence scores of interactions are scaled by the geometric mean of the binding partners’ expression percentiles. Due to the recomputed confidence scores, four interaction partners have been replaced by other proteins.

The protein–chemical network in STITCH is global and as such considers interactions anywhere in an organism. However, in multicellular organisms such as humans, not all proteins are present in every tissue. STITCH 5 addresses this through a new feature that allows users to filter a human interaction network so that only the proteins believed to be present in a specified tissue are shown (Figure 2). To provide this feature, STITCH now integrates tissue-specific protein expression patterns from two data sources. First, the TISSUES resource (33), which combines evidence from UniProt annotations, systematic large-scale transcriptomics and proteomics studies, and co-occurrence text mining. For use in STITCH, the text-mining evidence was recomputed based on the same texts used elsewhere in STITCH. Second, STITCH incorporates baseline expression patterns from tissues deposited in the Expression Atlas (34). Before augmenting the network with tissues data, users have to choose if they want to use data from TISSUES or Expression Atlas. The TISSUES resource contains confidence levels ranging from one (lowest confidence) to five (highest confidence). Accordingly, on the STITCH website users can select a tissue and a minimum confidence level. In contrast, datasets from the Expression Atlas are transformed into percentiles. The confidence score for a protein–protein interaction in the given tissue is then multiplied with the geometric mean of the two proteins’ expression percentiles. For protein–chemical interactions, the confidence score is multiplied with the protein's expression percentile. To access the tissue expression patterns, users can search for tissues either by typing parts of the tissue names or by selecting a tissue from a list. Then, users can submit the changed settings to STITCH. In return, an updated network will be shown. As non-expressed nodes are removed (using TISSUES) or confidence values get updated (using Expression Atlas), other interaction partners may become part of the network.

## USE CASES

STITCH has been widely used for a variety of different purposes. These fall into three broad classes: (i) small- to medium-scale analyses performed via the web interface, (ii) large-scale analyses that make use of the bulk download files and (iii) reuse of data from STITCH for development of new web-based resources.

Work by O'Reilly *et* *al*. on identifying potential drug targets for α1-antitrypsin deficiency exemplifies the web-based usage (35). Through a genome-wide RNAi screen in a *Caenorhabditis elegans* disease model, the authors identified 104 *C. elegans* genes of interest (having 85 human orthologs). To validate these as potential drug targets, the authors queried STITCH and MetaCore for each of the human proteins and thereby identified a compounds for use in follow-up experiments. Conversely, STITCH can also be queried for a set of chemicals to identify possible targets, as exemplified by the screen by Kumar *et* *al*. of compounds capable of altering intracellular manganese levels (36). The ability to see binding affinities in the new web interface makes STITCH 5 even better suited for such use cases than previous versions.

STITCH is also commonly used for large-scale analyses, which we facilitate by making the data available for bulk download. Ligeti *et* *al*. used these files to construct a network neighborhood of proteins around each drug and showed that the neighborhood overlap of two drugs can predict synergy of drug combinations (37). On a related note, Vogt *et* *al*. made use of both the drug thesaurus and the protein–chemical interaction from STITCH to predict drug contraindications (38).

Last, but not least, the integrated data provided by STITCH is useful to researchers who develop their own web resources and prediction methods. An example of this is the ChemDIS resource, which combines the protein–chemical interactions from STITCH with tools for gene enrichment analysis to link chemicals via proteins to GO terms, pathways and diseases (39). The experimental protein–chemical interactions from STITCH are also sometimes used as a benchmark set when developing prediction methods as exemplified by Zhou *et* *al*. (40).

## References

[1] Schwikowski B., Uetz P., Fields S. (2000). A network of protein–protein interactions in yeast. Nat. Biotechnol..

[2] Hishigaki H., Nakai K., Ono T., Tanigami A., Takagi T. (2001). Assessment of prediction accuracy of protein function from protein-protein interaction data. Yeast.

[3] Sharan R., Ulitsky I., Shamir R. (2007). Network-based prediction of protein function. Mol. Syst. Biol..

[4] Barabási A.-L., Gulbahce N., Loscalzo J. (2011). Network medicine: a network-based approach to human disease. Nat. Rev. Genet..

[5] Oti M., Snel B., Huynen M.A., Brunner H.G. (2006). Predicting disease genes using protein-protein interactions. J. Med. Genet..

[6] Yang K., Bai H., Ouyang Q., Lai L., Tang C. (2008). Finding multiple target optimal intervention in disease-related molecular network. Mol. Syst. Biol..

[7] Hopkins A.L. (2008). Network pharmacology: the next paradigm in drug discovery. Nat. Chem. Biol..

[8] Hopkins A.L., Groom C.R. (2002). The druggable genome. Nat. Rev. Drug Discov..

[9] Kalinina O.V., Wichmann O., Apic G., Russell R.B. (2012). ProtChemSI: a network of protein–chemical structural interactions. Nucleic Acids Res..

[10] Reddy A.S., Amarnath H.S.D., Bapi R.S., Sastry G.M., Sastry G.N. (2008). Protein ligand interaction database (PLID). Comput. Biol. Chem..

[11] Wassermann A.M., Bajorath J. (2011). BindingDB and ChEMBL: online compound databases for drug discovery. Expert Opin. Drug Discov..

[12] Kanehisa M., Goto S., Sato Y., Kawashima M., Furumichi M., Tanabe M. (2014). Data, information, knowledge and principle: back to metabolism in KEGG. Nucleic Acids Res..

[13] Chatr-Aryamontri A., Breitkreutz B.J., Heinicke S., Boucher L., Winter A., Stark C., Nixon J., Ramage L., Kolas N., O'Donnell L. (2013). The BioGRID interaction database: 2013 update. Nucleic Acids Res..

[14] Kjærulff S.K., Wich L., Kringelum J., Jacobsen U.P., Kouskoumvekaki I., Audouze K., Lund O., Brunak S., Oprea T.I., Taboureau O. (2013). ChemProt-2.0: visual navigation in a disease chemical biology database. Nucleic Acids Res..

[15] Hopkins A.L., Groom C.R., Alex A. (2004). Ligand efficiency: a useful metric for lead selection. Drug Discov. Today.

[16] Geeleher P., Cox N.J., Huang R.S. (2014). Clinical drug response can be predicted using baseline gene expression levels and in vitro drug sensitivity in cell lines. Genome Biol..

[17] Szklarczyk D., Franceschini A., Wyder S., Forslund K., Heller D., Huerta-Cepas J., Simonovic M., Roth A., Santos A., Tsafou K.P. (2015). STRING v10: protein–protein interaction networks, integrated over the tree of life. Nucleic Acids Res..

[18] Kuhn M., Szklarczyk D., Pletscher-Frankild S., Blicher T.H., von Mering C., Jensen L.J., Bork P. (2014). STITCH 4: integration of protein–chemical interactions with user data. Nucleic Acids Res..

[19] Law V., Knox C., Djoumbou Y., Jewison T., Guo A.C., Liu Y., Maciejewski A., Arndt D., Wilson M., Neveu V. (2014). DrugBank 4.0: shedding new light on drug metabolism. Nucleic Acids Res..

[20] Okuno Y., Tamon A., Yabuuchi H., Niijima S., Minowa Y., Tonomura K., Kunimoto R., Feng C. (2008). GLIDA: GPCR-ligand database for chemical genomics drug discovery—database and tools update. Nucleic Acids Res..

[21] Günther S., Kuhn M., Dunkel M., Campillos M., Senger C., Petsalaki E., Ahmed J., Urdiales E.G., Gewiess A., Jensen L.J. (2008). SuperTarget and Matador: resources for exploring drug-target relationships. Nucleic Acids Res..

[22] Zhu F., Shi Z., Qin C., Tao L., Liu X., Xu F., Zhang L., Song Y., Liu X., Zhang J. (2012). Therapeutic target database update 2012: a resource for facilitating target-oriented drug discovery. Nucleic Acids Res..

[23] Davis A.P., Grondin C.J., Lennon-Hopkins K., Saraceni-Richards C., Sciaky D., King B.L., Wiegers T.C., Mattingly C.J. (2015). The Comparative Toxicogenomics Database's 10th year anniversary: update 2015. Nucleic Acids Res..

[24] Schaefer C.F., Anthony K., Krupa S., Buchoff J., Day M., Hannay T., Buetow K.H. (2009). PID: the Pathway Interaction Database. Nucleic Acids Res..

[25] Croft D., Mundo A.F., Haw R., Milacic M., Weiser J., Wu G., Caudy M., Garapati P., Gillespie M., Kamdar M.R. (2014). The Reactome pathway knowledgebase. Nucleic Acids Res..

[26] Caspi R., Altman T., Billington R., Dreher K., Foerster H., Fulcher C.A., Holland T.A., Keseler I.M., Kothari A., Kubo A. (2014). The MetaCyc database of metabolic pathways and enzymes and the BioCyc collection of Pathway/Genome Databases. Nucleic Acids Res..

[27] Bento A.P., Gaulton A., Hersey A., Bellis L.J., Chambers J., Davies M., Krüger F.A., Light Y., Mak L., McGlinchey S. (2014). The ChEMBL bioactivity database: an update. Nucleic Acids Res..

[28] Roth B.L., Lopez E., Patel S., Kroeze W.K. (2000). The multiplicity of serotonin receptors: uselessly diverse molecules or an embarrassment of riches. Neuroscientist.

[29] Rose P.W., Prlić A., Bi C., Bluhm W.F., Christie C.H., Dutta S., Green R.K., Goodsell D.S., Westbrook J.D., Woo J. (2015). The RCSB Protein Data Bank: views of structural biology for basic and applied research and education. Nucleic Acids Res..

[30] Anastassiadis T., Deacon S.W., Devarajan K., Ma H., Peterson J.R. (2011). Comprehensive assay of kinase catalytic activity reveals features of kinase inhibitor selectivity. Nat. Biotechnol..

[31] Davis M.I., Hunt J.P., Herrgard S., Ciceri P., Wodicka L.M., Pallares G., Hocker M., Treiber D.K., Zarrinkar P.P. (2011). Comprehensive analysis of kinase inhibitor selectivity. Nat. Biotechnol..

[32] Franceschini A., Szklarczyk D., Frankild S., Kuhn M., Simonovic M., Roth A., Lin J., Minguez P., Bork P., von Mering C. (2013). STRING v9.1: protein-protein interaction networks, with increased coverage and integration. Nucleic Acids Res..

[33] Santos A., Tsafou K., Stolte C., Pletscher-Frankild S., O'Donoghue S.I., Jensen L.J. (2015). Comprehensive comparison of large-scale tissue expression datasets. PeerJ.

[34] Petryszak R., Burdett T., Fiorelli B., Fonseca N.A., Gonzalez-Porta M., Hastings E., Huber W., Jupp S., Keays M., Kryvych N. (2014). Expression Atlas update–a database of gene and transcript expression from microarray- and sequencing-based functional genomics experiments. Nucleic Acids Res..

[35] O'Reilly L.P., Long O.S., Cobanoglu M.C., Benson J.A., Luke C.J., Miedel M.T., Hale P., Perlmutter D.H., Bahar I., Silverman G.A. (2014). A genome-wide RNAi screen identifies potential drug targets in a C. elegans model of α1-antitrypsin deficiency. Hum. Mol. Genet..

[36] Kumar K.K., Lowe E.W. Jr, Aboud A.A., Neely M.D., Redha R., Bauer J.A., Odak M., Weaver C.D., Meiler J., Aschner M. (2014). Cellular manganese content is developmentally regulated in human dopaminergic neurons. Sci. Rep..

[37] Ligeti B., Pénzváltó Z., Vera R., Győrffy B., Pongor S. (2015). A network-based target overlap score for characterizing drug combinations: high correlation with cancer clinical trial results. PLoS One.

[38] Vogt I., Prinz J., Campillos M. (2014). Molecularly and clinically related drugs and diseases are enriched in phenotypically similar drug-disease pairs. Genome Med..

[39] Tung C.W. (2015). ChemDIS: a chemical-disease inference system based on chemical-protein interactions. J. Cheminform..

[40] Zhou H., Gao M., Skolnick J. (2015). Comprehensive prediction of drug-protein interactions and side effects for the human proteome. Sci. Rep..

